# Effect of Regulation of Chemerin/Chemokine-like Receptor 1/Stimulator of Interferon Genes Pathway on Astrocyte Recruitment to Aβ Plaques

**DOI:** 10.3390/ijms25084324

**Published:** 2024-04-13

**Authors:** Zhen Liu, Yijun Chen, Yanqing Chen, Jiayi Zheng, Wanning Wu, Linlin Wang, Hanqi Wang, Yang Yu

**Affiliations:** Engineering Research Center of Cell and Therapeutic Antibody Medicine, Ministry of Education, School of Pharmacy, Shanghai Jiao Tong University, Shanghai 200240, China; lz974639707@sjtu.edu.cn (Z.L.); chenyj98@sjtu.edu.cn (Y.C.); lorde_chen@sjtu.edu.cn (Y.C.); zjy111@sjtu.edu.cn (J.Z.); violawwn_9658@sjtu.edu.cn (W.W.); sjtu18842417985@sjtu.edu.cn (L.W.); wanghanqi@sjtu.edu.cn (H.W.)

**Keywords:** Alzheimer’s disease, astrocyte, migration, chemerin, CMKLR1, chemR23, STING, Aβ

## Abstract

Recruitment and accumulation of reactive astrocytes around senile plaques are common pathological features of Alzheimer’s disease (AD), with unclear mechanisms. Chemerin, an adipokine implicated in neuroinflammation, acts through its receptor, chemokine-like receptor 1 (CMKLR1), which also functions as a receptor for amyloid β (Aβ). The impact of the chemerin/CMKLR1 axis on astrocyte migration towards Aβ plaques is unknown. Here we investigated the effect of CMKLR1 on astrocyte migration around Aβ deposition in APP/PS1 mice with *Cmklr1* knockout (APP/PS1-*Cmklr1*^−/−^). CMKLR1-expressed astrocytes were upregulated in the cortices and hippocampi of 9-month-old APP/PS1 mice. Chemerin mainly co-localized with neurons, and its expression was reduced in the brains of APP/PS1 mice, compared to WT mice. CMKLR1 deficiency decreased astrocyte colocalization with Aβ plaques in APP/PS1-*Cmklr1*^−/−^ mice, compared to APP/PS1 mice. Activation of the chemerin/CMKLR1 axis promoted the migration of primary cultured astrocytes and U251 cells, and reduced astrocyte clustering induced by Aβ_42_. Mechanistic studies revealed that chemerin/CMKLR1 activation induced STING phosphorylation. Deletion of STING attenuated the promotion of the chemerin/CMKLR1 axis relative to astrocyte migration and abolished the inhibitory effect of chemerin on Aβ_42_-induced astrocyte clustering. These findings suggest the involvement of the chemerin/CMKLR1/STING pathway in the regulation of astrocyte migration and recruitment to Aβ plaques/Aβ_42_.

## 1. Introduction

Alzheimer’s disease (AD) is a chronic neurodegenerative disorder and the most common cause of dementia [1]. One of its primary pathological features is the presence of extracellular senile plaques composed of accumulated amyloid-β (Aβ) [2]. In recent decades, the development of disease-modifying therapies targeting Aβ has been a focal point of AD drug research. It is encouraging that two such drugs, aducanumab and lecanemab, have recently received accelerated approval from the US Food & Drug Administration for AD treatment, although further validation of their efficacy and long-term safety is required [3,4,5]. Aggregated Aβ has been proven to play a pivotal role in the activation of immune cells in the central nervous system (CNS), such as microglia and astrocytes. These cells induce neuroinflammation and are involved in the progression of AD [6,7,8]. Astrocytes are the most abundant neuroglial cells in the mammalian brain. Reactive astrocytes were found colocalized with and surrounding Aβ plaques in both AD patients and animal models [2]. Research indicates that disease-associated astrocytes exhibit both beneficial and detrimental functions in AD [8]. Reactive astrocytes have been shown to migrate towards Aβ deposition and degrade Aβ in vivo and in vitro [9]. Conversely, astrocytes can also be polarized into a pro-inflammatory phenotype induced by Aβ synergistically with tumor necrosis factor-α (TNF-α) and interleukin-1α (IL-1α), or in the presence of activated microglia [10,11,12]. Astrocytes of this phenotype lose their phagocytic capacity and produce various pro-inflammatory factors and neurotoxins, ultimately leading to neuronal death and AD progression [13]. Pro-inflammatory reactive astrocytes are found in large numbers in the brain tissue of individuals with AD [14]. Hence, elucidating the mechanism underlying astrocyte migration holds promise in providing new insight into the pathogenesis of AD and the development of therapeutic drugs.

Chemerin is an adipokine produced and released by different tissues and cells, including adipose tissue [15] and immune cells [16,17]. Three natural receptors have been found for chemerin, of which only chemokine-like receptor 1 (CMKLR1, also known as chemR23) is responsible for regulating immune cell inflammation and chemotactic responses [18,19,20,21]. CMKLR1, a G protein-coupled receptor, is expressed in hematopoietic tissues and immune cells such as macrophages, dendritic cells (DCs), and natural killer (NK) cells [22]. Studies have shown that the chemerin/CMKLR1 axis is related to inflammation, metabolism, and cancerogenesis [23,24]. Both chemerin and CMKLR1 have been detected in the mammalian brain [18,25]. Our study and other studies have found that CMKLR1 is expressed in neurons and glial cells and upregulates in post-mortem human brains with AD and AD animal models [25,26,27]. Emre et al. reported that CMKLR1 levels are associated with the activation of microglia and astrocytes and positively correlate with the Braak stages in AD [27]. Our previous studies have shown that CMKLR1 is also a functional receptor for Aβ [25]. Both Aβ and chemerin induce microglial migration after binding to CMKLR1 [25,28]. CMKLR1 deletion reduces mortality and improves the cognitive impairment of AD mice [26]. Additionally, studies have shown that CMKLR1 agonists resolvin E1 (RvE1) and chemerin-9 (C9, derived from chemerin) attenuate oxidative stress and suppress NLRP3 inflammasome activation through the CMKLR1/Nrf2/TXNIP pathway, leading to cognitive improvement in diabetic mice [29]. Research also found that chemerin ameliorates hypoxic–ischemic-induced neuronal apoptosis and promotes M2 microglia in neonatal rat germinal matrix hemorrhage (GMH) models via the CMKLR1/CAMKK2/AMPK pathway [30,31]. These findings indicate that the chemerin/CMKLR1 axis could participate in the pathological process of AD by regulating neuroinflammation. The impact of this axis on astrocyte function as migration has not yet been investigated.

The stimulator of interferon genes (STING), an endoplasmic reticulum adaptor, is involved in the innate immune signaling which provides a defense against pathogens [32]. STING and its upstream component (cyclic GMP-AMP synthase, cGAS) are upregulated in the brains of individuals with AD and AD mouse models [33,34]. Studies have shown that they are constitutively expressed in microglia and astrocytes [35]. Research indicates that the cGAS-STING pathway in glial cells contributes to neuroinflammation [33,34,36,37]. Deletion of cGAS in microglia suppresses the pro-inflammatory astrocytic phenotype and attenuates oligomeric Aβ-induced neurotoxicity [33]. Activation of the astrocytic STING pathway results in the production of inflammatory cytokines such as interferon-α (IFN-α) and TNF [37]. The STING pathway has been implicated in the migration of Ly6C^hi^ monocytes [38], fibroblast-like synoviocytes [39], and gastric cancer cells [40]. However, the impact of the STING pathway on glial cell migration remains unknown.

This study postulates that the chemerin/CMKLR1 axis contributes to astrocyte migration and recruitment to Aβ plaques. We examined the expression of CMKLR1 in astrocytes from APP/PS1 double-transgenic mice. Then we explored the effect of CMKLR1 on the migration of astrocytes to Aβ deposition in APP/PS1 double-transgenic mice with *CMKLR1* knockout (APP/PS1-*Cmklr1*^−/−^) generated by crossing APP/PS1 mice with *Cmklr1*^−/−^ mice. CMKLR1-expressed astrocytes were increased in APP/PS1 mice compared to WT littermates. CMKLR1 deficiency suppressed the migration and recruitment of astrocytes toward Aβ deposition in APP/PS1-*Cmklr1*^−/−^ mice compared to APP/PS1 mice. Mechanism investigations revealed that activation of the chemerin/CMKLR1 axis promoted the migration of primary astrocytes from WT mice but not from *Sting* knockout (*Sting*^−/−^) mice, suggesting the involvement of the STING pathway in chemerin/CMKLR1-induced astrocyte migration. Furthermore, chemerin suppressed Aβ-induced astrocyte aggregation. STING deficiency alleviated the inhibition of chemerin on Aβ-induced astrocyte accumulation. These findings provide insight into the role of the chemerin/CMKLR1/STING pathway in astrocyte migration and recruitment to Aβ plaques/Aβ_42_.

## 2. Results

### 2.1. Elevated CMKLR1-Expressed Astrocytes in the Brain of APP/PS1 Mice

**Figure 1:**
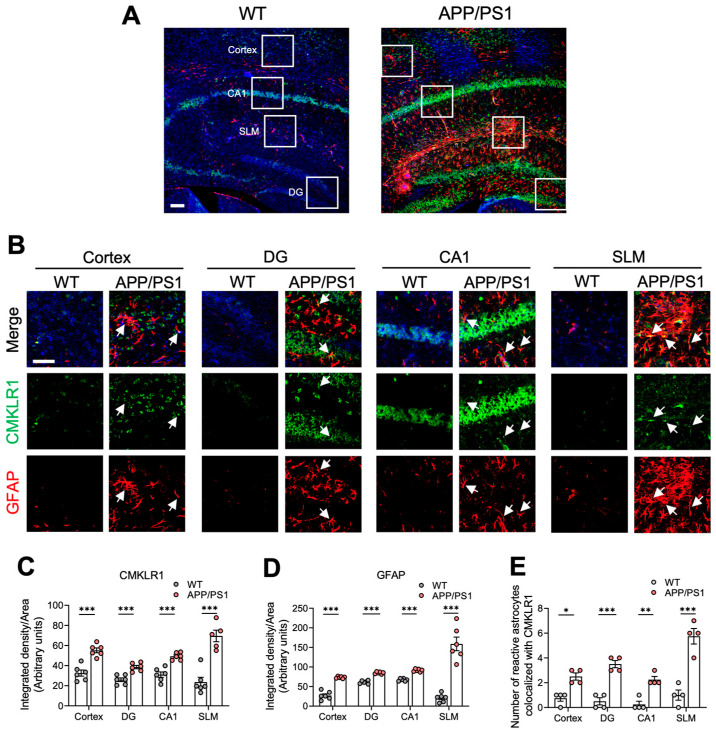
Increases of astrocyte-expressed CMKLR1 in the cortices and hippocampi of APP/PS1 transgenic mice. (**A**) Immunofluorescence staining was conducted to detect the expression of CMKLR1 in astrocytes. Serial sections of WT and APP/PS1 mouse brains at 9 months of age were stained for CMKLR1 using a rabbit anti-CMKLR1/AF488 antibody (green fluorescence) and astrocytic marker GFAP using a mouse anti-GFAP-Cy3^TM^ antibody (red fluorescence). Cell nuclei were stained with DAPI (blue fluorescence). Scale bar, 100 μm. (**B**) Selected areas of the cortex and DG, CA1, and SLM regions of the hippocampus in panel A are magnified six times and presented as combined and individual fluorescence stains. Scale bar, 25 μm. The white arrows indicate the colocalization of CMKLR1 and GFAP in the cortices and hippocampi in APP/PS1 mice. Quantification of the fluorescence intensities of CMKLR1 and GFAP and the number of their colocalization are shown in (**C**), (**D**), and (**E**), respectively. The results are expressed as the mean ± SEM based on 2 individual fields for each region, using three mice in each group. * *p* < 0.05, ** *p* < 0.01, *** *p* < 0.001. DG: dentate gyrus, CA1: cornu ammonis-1, SLM: stratum lacunosum-moleculare.

Our previous studies have shown that CMKLR1 participates in the AD pathological process by mediating neuronal tau propagation [26] and microglia migration [28]. Here we further explored the role and mechanism of the astrocyte-expressed CMKLR1 in AD pathology. We first examined the expression of CMKLR1 and its colocalization with astrocytes in the brains of 9-month-old APP/PS1 transgenic mice. Immunofluorescence staining was conducted with anti-CMKLR1 and anti-glial fibrillary acidic protein (GFAP) antibodies to detect CMKLR1 and astrocytes, respectively. As shown in Figure 1A–D, significantly elevated expressions of CMKLR1 (green fluorescence) and GFAP (red fluorescence) were observed in the cortex as well as in the dentate gyrus (DG), cornu ammonis-1 (CA1), and stratum lacunosum-moleculare (SLM) regions of the hippocampus of APP/PS1 mice, compared to findings in WT littermates. Furthermore, the number of astrocytes co-localized with CMKRL1 (yellow fluorescence) increased markedly in these brain regions, especially the SLM region of the hippocampus, in APP/PS1 mice, compared to WT mice (Figure 1E).

### 2.2. The Chemerin/CMKLR1 Axis Is Weakened in APP/PS1 Mice

**Figure 2:**
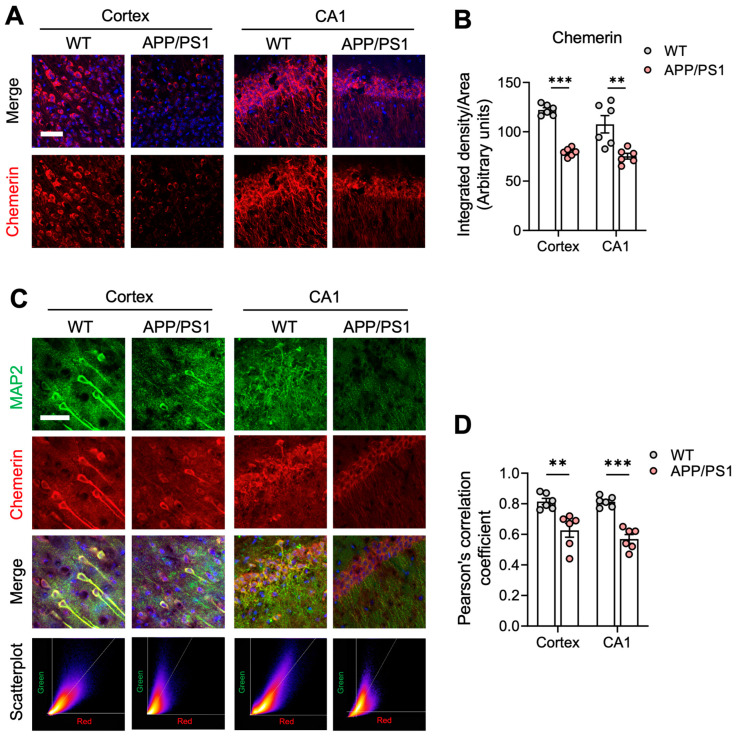
Reduced chemerin expression and neuronal colocalization in the brains of APP/PS1 transgenic mice. (**A**) Immunofluorescence staining was conducted to detect the expression of chemerin. Serial sections in the cortices and CA1 regions of hippocampi from 9-month-old WT and APP/PS1 mice were stained for chemerin using a rabbit anti-chemerin/AF647 antibody (red fluorescence). Cell nuclei were stained with DAPI (blue fluorescence). Z-stack confocal images were acquired and merged into maximum intensity projections (chemerin and DAPI). Scale bar, 50 μm. (**B**) Quantification of the fluorescence intensity of chemerin is shown. (**C**) Colocalization of chemerin (red fluorescence) with neuronal marker MAP2 (using a rabbit anti-MAP2 polyclonal antibody and Alexa fluor 488-conjugated anti-rabbit IgG, green fluorescence) in the cortices and CA1 regions of WT and APP/PS1 mice aged 9 months. Cell nuclei were stained with DAPI (blue fluorescence). Representative scatter plots showing pixel intensities in the red channel versus the green channel are displayed below each merged image, with Pearson’s correlation coefficients of r = 0.87, 0.67 in the cortices of WT and APP/PS1 mice, respectively, and 0.84 and 0.62 in the CA1 region. Scale bar, 50 μm. (**D**) Pearson’s correlation coefficient was utilized to quantify the colocalization of chemerin and MAP2. Image J/Fiji software 1.54f was used to calculate the coefficient. The results are expressed as the mean ± SEM based on 2 individual fields for each region, using three mice in each group. ** *p* < 0.01, *** *p* < 0.001. CA1: cornu ammonis-1.

We further tested the expression of chemerin, the endogenous ligand of CMKLR1, in AD mouse brains. Compared to WT mice, chemerin was significantly reduced in APP/PS1 mouse brains (Figure 2A,B, Supplementary Figure S1). To characterize the cellular distribution of chemerin, double immunofluorescence staining was conducted for chemerin and MAP2 (the neuron-specific molecular). Scatter plots generated from the images demonstrated strong co-localization between chemerin and MAP2, especially in the CA1 regions and cortices of wild-type mice (Figure 2C,D). Chemerin showed significantly less co-localization with neurons, as indicated by MAP2, in the CA1 and cortex of APP/PS1 mice compared to wild-type mice (Figure 2C,D). Additionally, the same trend was observed in the DG and SLM of the hippocampus (Supplementary Figure S2). This result indicates transcellular regulation of the chemerin/CMKLR1 axis in the brain. Additionally, Pearson correlation coefficients between chemerin and MAP2 expression revealed that chemerin was predominantly co-localized with neurons in WT mice (Figure 2D), indicating that neuronally derived chemerin may target astrocyte-expressed CMKLR1 to exert immune functions in the CNS. We have shown that Aβ is another endogenous ligand of CMKLR1 [25], suggesting that the Aβ/CMKLR1 axis is enhanced in AD pathology. These findings suggest the imbalance of the chemerin/CMKLR1 and Aβ/CMKLR1 axes in AD mice, and astrocyte-expressed CMKLR1 may contribute to the Aβ pathology.

### 2.3. CMKLR1 Deficiency Reduces the Colocalization of Astrocytes with Aβ Plaques in the Brain of APP/PS1 Mice

**Figure 3:**
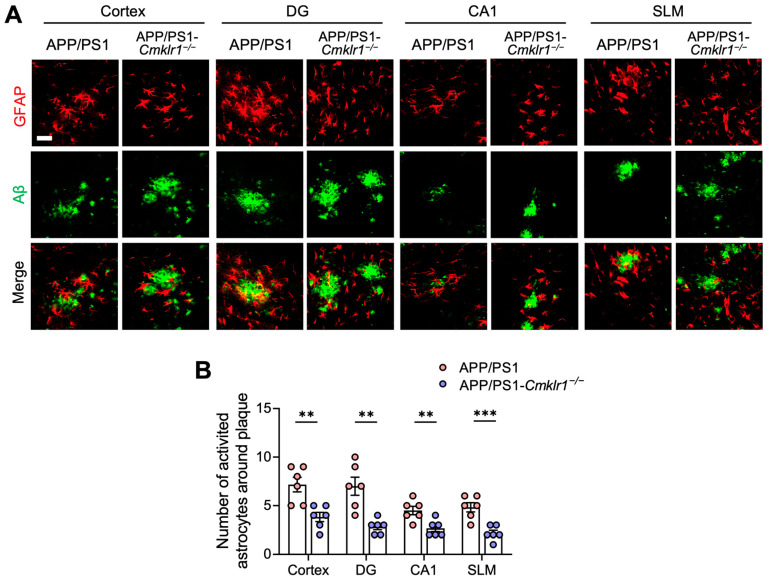
CMKLR1 deficiency decreases the number of reactive astrocytes around Aβ plaques in the cortices and hippocampi of APP/PS1 transgenic mice. (**A**) Immunofluorescence staining was conducted to detect reactive astrocytes around Aβ plaques. Serial sections of the cortex and DG, CA1, and SLM regions of the hippocampus from 9-month-old APP/PS1 and APP/PS1-*Cmklr1*^−/−^ mice were stained for Aβ plaques using a rabbit anti-Aβ antibody and Alexa Fluor 647-conjugated anti-rabbit IgG (shown as green fluorescence) and GFAP using a mouse anti-GFAP-Cy3^TM^ antibody (red fluorescence). Scale bar, 50 μm. (**B**) Quantification of the number of astrocytes surrounding Aβ plaques is presented. The results are expressed as the mean ± SEM based on 2 individual fields for each region, using three mice in each group. ** *p* < 0.01, *** *p* < 0.001. DG: dentate gyrus, CA1: cornu ammonis-1, SLM: stratum lacunosum-moleculare.

CMKLR1 participates in immune responses and has the properties of chemokine receptors [18]. We sought to determine whether CMKLR1 contributes to the recruitment of astrocytes toward Aβ plaques in AD mice. We generated APP/PS1 mice with CMKLR1 deficiency (APP/PS1-*Cmklr1*^−/−^ mice) by crossing APP/PS1 mice with *Cmklr1*^−/−^ mice [26], both on a C57BL/6J background. Double immunofluorescence staining for GFAP and Aβ plaques was performed. Astrocytes (red fluorescence) within 25 µm of an Aβ plaque (green fluorescence) were considered co-localized [41]. A significant reduction in the number of reactive astrocytes co-localized with Aβ deposits was observed in the cortices and hippocampi of APP/PS1-*Cmklr1*^−/−^ mice, compared to APP/PS1 mice (Figure 3A,B), suggesting that CMKLR1 deficiency suppressed astrocyte migration and aggregation towards Aβ deposits. These findings indicate that astrocyte-expressed CMKLR1 may participate in AD pathology by regulating the recruitment and migration of astrocytes to Aβ.

### 2.4. Activation of the Chemerin/CMKLR1 Axis Induces Astrocyte Migration

**Figure 4:**
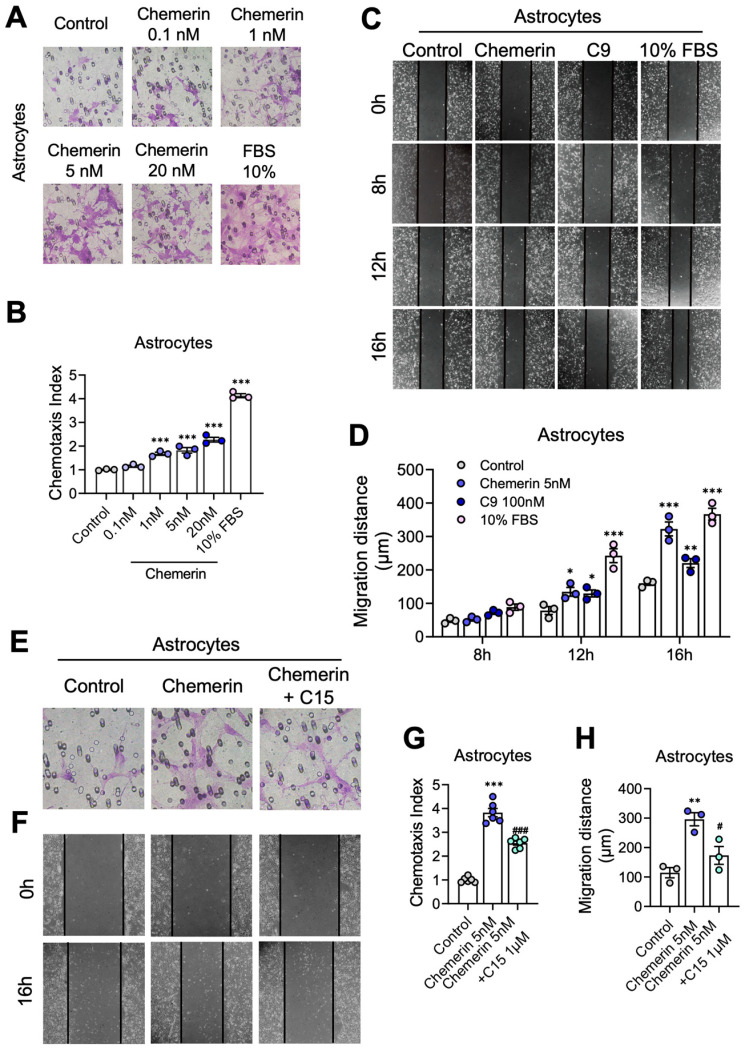
Activation of the chemerin/CMKLR1 axis induces the migration of primary astrocytes. (**A**) The Boyden chamber migration assay was conducted to detect cell migration. Primary cultures of astrocytes were treated with chemerin (0.1–20 nM) or 10% FBS for 12 h. Representative images of migrated astrocytes on membrane filters are shown. Magnification, 400×. (**B**) Quantification of the degree of increase in the number of migrated cells in response to chemerin or 10% FBS over the control medium is shown. (**C**) Primary cultures of astrocytes were treated with 5 nM chemerin, 100 nM C9, or 10% FBS, and cell migration was detected by the scratch-wound assay. The astrocytes were photographed at 0 h, 8 h, 12 h, and 16 h. Representative images of migrated astrocytes are shown. Magnification, 100×. (**D**) Quantification of the migration distance is shown. (**E**,**F**) Primary astrocytes were incubated with chemerin with or without a 15-min pretreatment of C15 (1 μM). The migration of astrocytes was detected by Boyden chamber and scratch-wound assays after 12 and 16 h incubation, respectively. Representative images of migrated astrocytes are shown. Magnification, 400× and 100×, respectively. Quantified data are shown in (**G**,**H**). The results are expressed as the mean ± SEM based on three independent experiments, each in triplicate. * *p* < 0.05, ** *p* < 0.01, *** *p* < 0.001, ^#^ *p* < 0.05, ^###^ *p* < 0.001; * compared to the control group, # compared to the group treated with chemerin.

First, the Boyden chamber assay was conducted to explore the effect of chemerin/CMKLR1 on the migration of astrocytes. Primary cultures of mouse astrocytes and human glioma U251 cells were seeded to the upper chamber wells, and chemerin (0.1–20 nM) was added to the lower wells as the chemoattractant. The cells that migrated through the filter were counted after 12 h. Chemerin significantly induced the migration of primary astrocytes and U251 cells in a dose-dependent manner (Figure 4A,B, and Supplementary Figure S3A,B). The treatment of C9 (an agonist of CMKLR1 derived from chemerin) and FBS (10%, as a positive control) also promoted the migration of primary astrocytes and U251 cells (Supplementary Figure S4A–C). Additionally, the MTT assay was performed to detect the effects of chemerin and C9 on the viability of primary astrocytes and U251 cells. The results showed that treatments of chemerin (20 nM) or C9 (100 nM) for 16 h did not affect the viability of these cells (Supplementary Figure S5A,B).

The scratch-wound assay was also performed to verify the promotion effect of chemerin on astrocyte migration. Primary cultured astrocytes and U251 cells were treated with 5 nM chemerin, 100 nM C9, or 10% FBS. After 8 h, 12 h, and 16 h, images were taken, and wound closure was quantified. A marked promotion of wound closure was observed in primary astrocytes and U251 cells treated with chemerin, C9, or 10% FBS compared to untreated controls (Figure 4C,D, and Supplementary Figure S3C,D), consistent with the results of the Boyden chamber assay. All of these results suggest that chemerin induces the migration of astrocytes.

Furthermore, to verify that the chemerin-induced migration of astrocytes is via CMKLR1, C15, a chemerin-derived 15-residue peptide lacking chemerin agonistic activity, was used [42]. The pre-treatment of C15 inhibited astrocyte migration induced by chemerin significantly in the Boyden chamber and scratch-wound assays (Figure 4E–H), indicating that the activation of the chemerin/CMKLR1 axis contributes to the migration of astrocytes.

### 2.5. Chemerin Suppresses Aβ-Induced Aggregation of Astrocytes

**Figure 5:**
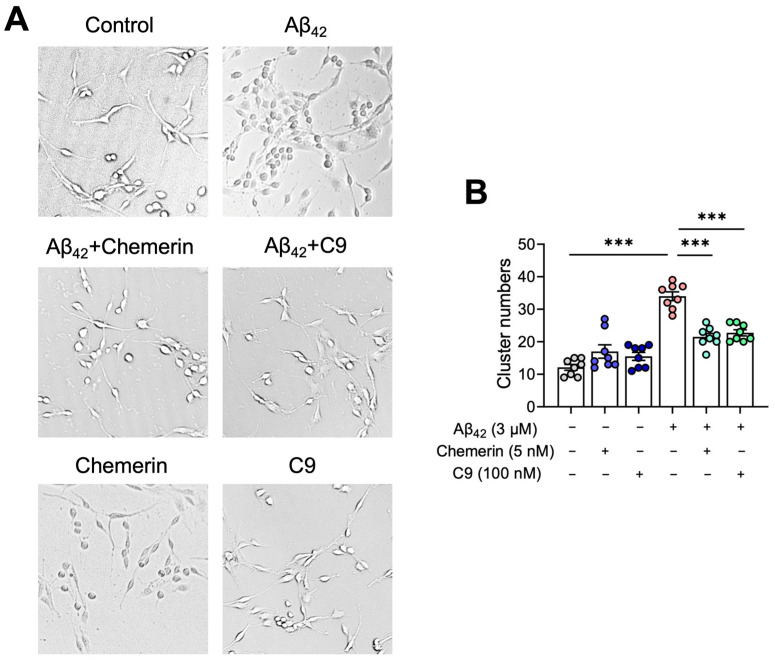
Activation of the chemerin/CMKLR1 axis reduces Aβ-induced astrocyte aggregation. (**A**) U251 cells were incubated with aggregated Aβ_42_ (3 μM) with or without pretreatments of chemerin (5 nM) or C9 (100 nM). Representative images of control, Aβ_42_, and Aβ_42_ with pretreatment of chemerin or C9 incubation. Magnification, 400×. (**B**) Quantification of the number of astrocyte aggregations is shown. The results are expressed as the mean ± SEM based on three independent experiments, each in triplicate. *** *p* < 0.001.

We next explored the effects of chemerin on the recruitment and aggregation of astrocytes towards Aβ in vitro. U251 cells were incubated with Aβ_42_ (3 μM) for 24 h, with and without a 15 min pre-treatment of chemerin (5 nM) or C9 (100 nM). As shown in Figure 5A,B, Aβ_42_ induced the aggregation of U251 cells, which was significantly inhibited by the treatment of chemerin or C9. These results suggest that chemerin/CMKLR1 could suppress the Aβ-induced accumulation of astrocytes. 

### 2.6. STING Contributes to Astrocyte Migration Induced by Chemerin/CMKLR1 Axis

**Figure 6:**
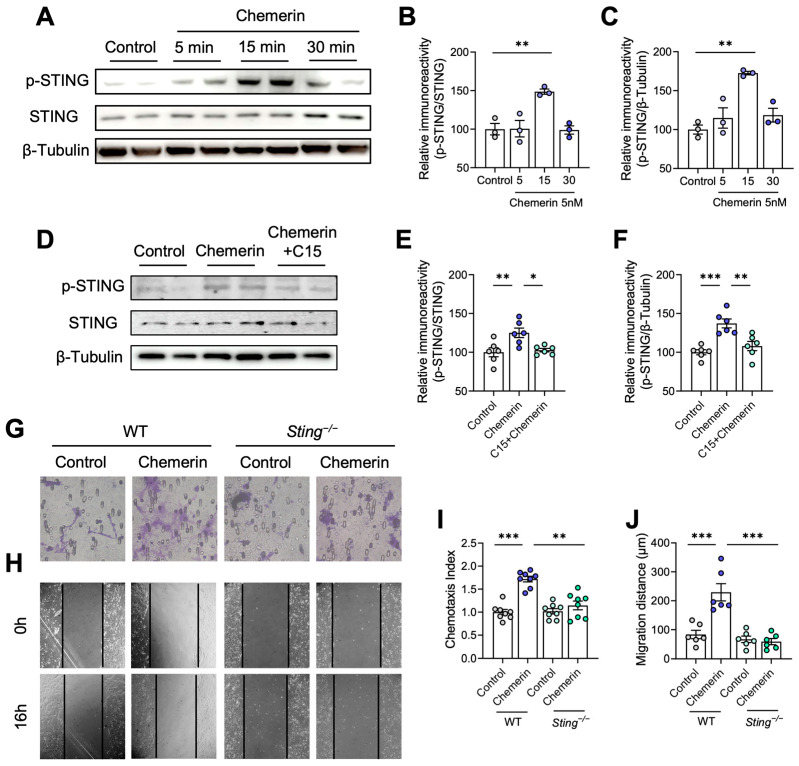
Chemerin induces astrocyte migration by activating the CMKLR1/STING pathway. (**A**) Primary cultures of astrocytes were incubated with 5 nM chemerin for 0, 5, 15, and 30 min. Representative Western blots show phosphorylated STING (p-STING) and STING. (**B**,**C**) Quantification of the immunoreactivity of the blots, normalized against STING or β-tubulin, respectively. (**D**) Primary astrocytes were incubated with 5 nM chemerin for 15 min with or without pretreatment of 1 μM C15. Representative Western blots show the expression of p-STING and STING. (**E**,**F**) Quantification of the immunoreactivity of the blots, normalized against STING or β-tubulin, respectively. (**G**,**H**) Primary astrocytes from WT and *Sting*^−/−^ mice were incubated with 5 nM chemerin. The migration of astrocytes was detected by Boyden chamber and scratch-wound assay after 12 and 16 h incubation. Quantified data are shown in (**I**,**J**). Magnification, 400× and 100×, respectively. The results are expressed as the mean ± SEM based on three independent experiments, each in triplicate. * *p* < 0.05, ** *p* < 0.01, *** *p* < 0.001.

Previous studies have shown that STING is involved in cell migration [38,43]. Here, we determined whether STING participates in the migration of astrocytes induced by the chemerin/CMKLR1 axis. The Western blot assay was first performed to test the effect of chemerin on the phosphorylation of STING. Primary cultured astrocytes were incubated with 5 nM chemerin for 5, 15, and 30 min. The results showed that 15 min of chemerin incubation significantly increased the phosphorylation of STING (Figure 6A–C), indicating that chemerin induces STING activation. Next, we examined whether chemerin induces the activation of STING through CMKLR1. Primary cultured astrocytes were incubated with 5 nM chemerin for 15 min with or without pre-treatment of C15 (1 μM). As shown in Figure 6D–F, C15 pre-treatment inhibited the chemerin-induced increase in STING phosphorylation. These findings suggest that STING is downstream of CMKLR1, and chemerin can activate the CMKLR1/STING signaling pathway in astrocytes.

We then explored whether the migration of astrocytes induced by chemerin/CMKLR1 is dependent on STING activation. The Boyden chamber and scratch-wound assays were conducted using primary astrocytes obtained from STING knockout (*Sting*^−/−^) and WT mice. The results showed that treatment with chemerin (5 nM) enhanced the migration of astrocytes from WT mice, while no effect was observed on astrocytes from the *Sting*^−/−^ mice (Figure 6G–J), suggesting that STING deficiency eliminated the promotion of chemerin-induced migration of astrocytes. These findings imply that STING plays a crucial role in regulating chemerin/CMKLR1-mediated astrocyte migration.

### 2.7. STING Contributes to the Inhibitory Effect of Chemerin on Aβ-Induced Aggregation of Astrocytes

**Figure 7:**
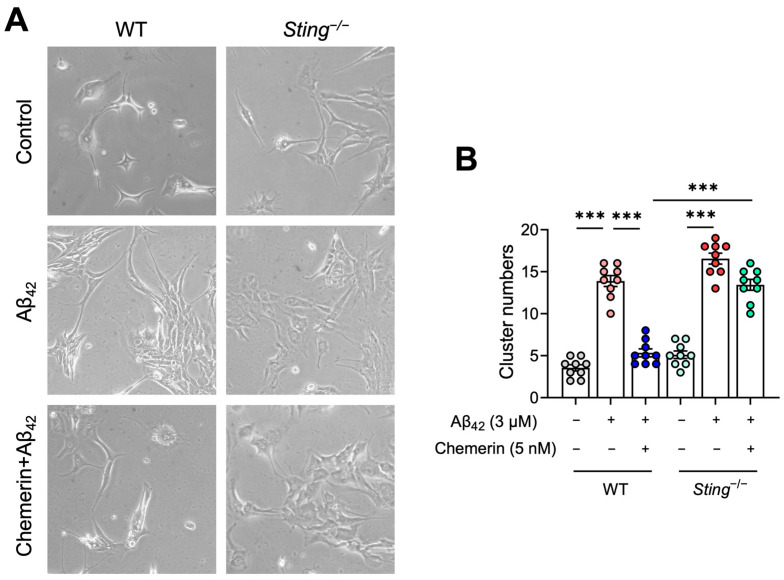
STING deficiency attenuates inhibition of the chemerin/CMKLR1 axis relative to Aβ-induced astrocyte aggregation. (**A**) Primary cultures of astrocytes from WT and *Sting*^−/−^ mice were incubated with aggregated Aβ_42_ (3 μM) with or without pretreatment of chemerin (5 nM). Representative images of control, Aβ_42_, and Aβ_42_ with pretreatment of chemerin incubation. Magnification, 400×. Quantified data is shown in (**B**), each group is represented by distinct colored dots. The results are expressed as the mean ± SEM based on three independent experiments, each in triplicate. *** *p* < 0.001.

Since we have confirmed that the chemerin/CMKLR1 axis is involved in the Aβ-induced migration and aggregation of astrocytes, we further attempted to detect whether STING is required for this effect. Primary cultured astrocytes from *Sting*^−/−^ and WT mice were incubated with Aβ_42_ (3 μM) for 24 h, with and without a 15 min pre-treatment of chemerin (5 nM). As shown in Figure 7A,B, Aβ induced the aggregation of primary astrocytes from the WT mouse brain, while chemerin treatment suppressed the accumulation of astrocytes induced by Aβ. In comparison, STING deficiency abrogated the suppressive effect of chemerin on astrocyte aggregation induced by Aβ. These findings suggest that the chemerin/CMKLR1/STING pathway contributes to Aβ-induced astrocyte aggregation.

## 3. Discussion

**Figure 8:**
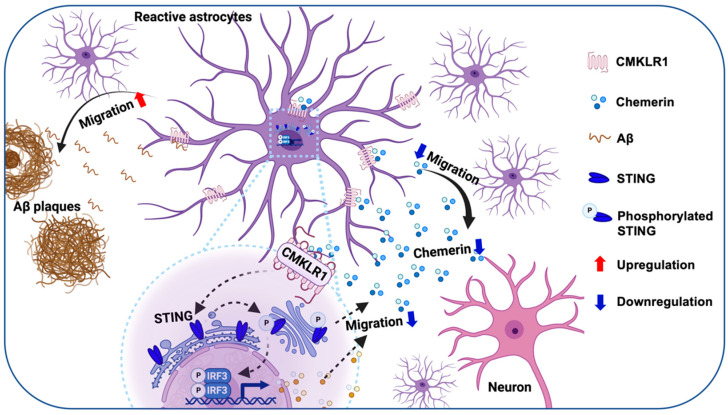
Schematic diagram showing the contribution of the chemerin/CMKLR1/STING pathway in the recruitment of astrocytes toward Aβ plaques, created with BioRender.com. Upregulation of CMKLR1 and imbalance of the chemerin/CMKLR1 and Aβ/CMKLR1 axes result in the recruitment of astrocytes to Aβ plaques in the AD pathological progression. An enhanced chemerin/CMKLR1 axis promotes astrocyte migration and reduces the accumulation and recruitment to Aβ. STING pathway activation contributes to the promotion of chemerin/CMKLR1 relative to astrocyte migration.

The recruitment and accumulation of reactive astrocytes around Aβ plaques are crucial pathological features of AD [2]. However, the underlying mechanisms of these pathological events remain unclear. The present study illustrates, for the first time, that the chemerin/CMKLR1 axis contributes to the migration and accumulation of astrocytes to Aβ deposition, both in vivo and in vitro. Both chemerin and Aβ are endogenous ligands for CMKLR1. Our present and previous studies [25] have shown that they induce astrocyte migration through the activation of CMKLR1. The present study found that CMKLR1-expressed astrocytes are upregulated, whereas chemerin in neurons is downregulated in APP/PS1 mice, suggesting that the imbalance of chemerin/CMKLR1 and Aβ/CMKLR1 axes in astrocytes results in the migration and accumulation of astrocytes toward Aβ plaques in AD pathological process (Figure 8). Our recent study has shown that the chemerin/CMKLR1 axis is also involved in microglial migration [28]. It is proposed that intercellular crosstalk between microglia, astrocytes, and neurons induces inflammatory dysregulation and feedback loops in neurodegenerative diseases, leading to an exacerbated inflammatory milieu and neuronal dysfunction that promotes disease progression [13,14]. Based on the results of double immunofluorescence staining, chemerin is predominantly expressed in neurons, indicating its potential role as a mediator of neuron-astrocyte/microglia interactions under both physiological and AD pathological conditions. These findings imply that the modulation of the chemerin/CMKLR1 axis could be a promising therapeutic strategy for AD. Our previous studies have shown that CMKLR1 expression in neurons contributes to tau propagation [26]. Further validation is needed to explore the effects of the chemerin/CMKLR1 axis in neurons on Aβ and tau pathology.

Moreover, this study is the first to demonstrate the involvement of the STING pathway in astrocyte migration, a pathway which is dependent on the chemerin/CMKLR1 axis. Previous studies have shown that activation of the chemerin/CMKLR1 axis triggers chemotactic responses in a variety of immune and non-immune cells [44,45,46,47,48]. This axis regulates the migration of smooth muscle cells (SMCs) through autophagy or the extracellular signal-regulated kinase (ERK) pathway [47,49]. Our recent study has shown that activation of the chemerin/CMKLR1 axis promotes microglial migration and polarization via the p38 mitogen-activated protein kinase (MAPK) pathway. The regulation of this pathway on the STING pathway has not been documented.

The cGAS-STING pathway has been implicated in neuroinflammation by its regulation of the immune functions of glial cells in several disease models, including neurodegenerative diseases (such as Alzheimer’s disease) [33,34,50,51], major depressive disorder (MDD) [52], experimental autoimmune encephalomyelitis (EAE) [36], ischemia/reperfusion-induced or traumatic brain injury [53,54,55], and brain metastasis [37]. The involvement of the STING pathway in the migration of Ly6C^hi^ monocytes [38], fibroblast-like synoviocytes [39], and gastric cancer cells [40] has been demonstrated. Limited studies have reported its effect on microglia migration, while its impact on astrocytes remains undocumented. Jin et al. revealed that the tau protein activates microglia via the polyglutamine binding protein 1 (PQBP1)-cGAS-STING pathway, leading to enhanced neuroinflammatory responses. Notably, PQBP1 deletion does not affect microglia migration or phagocytosis [50]. Additionally, another study reported that A151, a cGAS antagonist, inhibits microglial proliferation and migration towards injured tissue, indicating the involvement of the cGAS-STING pathway in microglial migration. Inhibition of cGAS can alleviate neuroinflammatory responses by reducing the infiltration of periphery neutrophils into the CNS [55]. Deletion of cGAS in microglia inhibits pro-inflammatory reactive astrocytes and alleviates oligomeric Aβ-induced neurotoxicity [33]. Limited studies have addressed the impact of the cGAS-STING pathway on astrocytic function. One study has documented that activation of the astrocytic STING pathway induces the release of inflammatory cytokines like IFN-α and TNF [37], highlighting its involvement in astrocyte-driven neuroinflammation. Further validation is needed to elucidate the precise mechanism by which this pathway influences astrocyte migration.

In conclusion, the present study illustrates the effect of the chemerin/CMKLR1 axis on astrocyte migration. Dysregulation of the chemerin/CMKLR1 and Aβ/CMKLR1 axes in AD affects the recruitment and migration of astrocytes to Aβ plaques, potentially contributing to the pathogenesis of AD. Modulating the chemerin/CMKLR1 axis to counteract overactivation of the Aβ/CMKLR1 axis is a promising strategy for AD treatment. Mechanistic investigations demonstrate that the STING pathway is involved in the effect of the chemerin/CMKLR1 axis on astrocyte migration. The chemerin/CMKLR1/STING pathway is involved in regulating the migration and accumulation of astrocytes to Aβ deposition/Aβ_42_.

## 4. Materials and Methods

### 4.1. Antibodies and Reagents

Dulbecco’s modified Eagle’s medium (DMEM), trypsin-ethylenediaminetetraacetic acid (trypsin-EDTA), and fetal bovine serum (FBS) were obtained from Gibco (Invitrogen, Carlsbad, CA, USA). Chemerin (recombinant human chemerin) was purchased from R&D systems (Minneapolis, MN, USA). The BCA protein assay kit and 4,6-diamidino-2-phenylindole (DAPI) were obtained from the Beyotime Institute of Biotechnology (Nantong, China). Chemerin9 (C9), chemerin15 (C15) peptides, and Aβ_42_ (≥95% purity) were synthesized at Shanghai Science Peptide Biologic Technology Co., Ltd. (Shanghai, China). Mouse monoclonal anti-GFAP-Cy3™ (catalog No. C9205) and rabbit polyclonal anti-MAP2 (catalog No. M3696) were obtained from Sigma-Aldrich, Inc. (St. Louis, MO, USA). Rabbit polyclonal anti-CMKLR1/AF488 (catalog No. BS-20043R) and anti-chemerin/AF647 antibodies (catalog No. BS-10410R) were purchased from Beijing Biosynthesis Biotechnology Co., Ltd. (Beijing, China). Rabbit polyclonal anti-Aβ (catalog No. 14975), anti-β-tubulin (catalog No. 2128), rabbit monoclonal anti-STING (catalog No. 13647), and anti-phospho-STING (catalog No. 72971) antibodies were from Cell Signaling Technology (Danvers, MA, USA). Rabbit and mouse polyclonal HRP-labeled IgG (catalog No. AW-WB1067 and AW-WB1064, respectively) were purchased from ALLWIN Biotechnology Co., Ltd. (Shanghai, China). AlexaFluor-488-conjugated anti-rabbit IgG secondary antibody (catalog No. A21206) was purchased from Invitrogen. AlexaFluor-647-conjugated anti-rabbit IgG secondary antibody (catalog No. Ab150079) was from Abcam (Cambridge, UK). Other reagents were purchased from Sigma-Aldrich.

### 4.2. Animals

The CMKLR1 knockout (*Cmklr1*^−/−^) mice in C57BL/6J background were generated by BRL Medicine Inc. (Shanghai, China). The STING knockout (*Sting*^−/−^, obtained from GemPharmatech, Nanjing, China) mice in C57BL/6J background were kindly provided by Dr. Ao Zhang and Dr. Chunyong Ding (Shanghai Jiao Tong University, Shanghai, China). The APP/PS1 double transgenic mice in C57BL/6J background (APP_SWE_/PS1ΔE9^+/−^, stock number 005864) were purchased from the Jackson Laboratory (Bar Harbor, ME, USA). All mice were group-housed (4–5 mice per cage) under a 12/12 h light and dark cycle, with free access to food and water. The housing, breeding, and animal experiments were conducted in accordance with the National Institutes of Health Guide for the Care and Use of Laboratory Animals, and approved by the Biological Research Ethics Committee of Shanghai Jiao Tong University (Protocol number: 202101323/Approval date: 14 October 2021). The breeding strategy involved crossing *Cmklr1*^−/−^ mice with APP/PS1 mice to generate APP/PS1-*Cmklr1*^+/−^ mice. Subsequently, these offspring were further bred with *Cmklr1*^+/−^ mice to establish three experimental groups: WT (APP/PS1^−/−^-*Cmklr1*^+/+^), APP/PS1 (APP/PS1^+/−^-*Cmklr1*^+/+^), and APP/PS1-*Cmklr1*^−/−^ (APP/PS1^+/-^-*Cmklr1*^−/−^) [26]. Genotypes of mice were examined using PCR. At 9 months of age, male and female mice from all three groups were euthanized by decapitation, and brains were promptly removed. The right hemispheres were fixed in 4% paraformaldehyde in 0.1 M phosphate-buffered saline (PBS) and cryoprotected in 30% sucrose. Coronal brain sections of 30 μm thickness were obtained using a freezing sliding microtome and stored in a glycol anti-freeze solution at −20 °C until immunofluorescence staining. The cerebral cortices and hippocampi of the left hemispheres were dissected, flash-frozen in dry ice, and stored at −80 °C for Western blot analysis.

### 4.3. Astrocyte Cultures

The primary astrocyte cultures were prepared from 1-day-old WT or *Sting*^−/−^ mouse pups in C57BL/6J background, as previously described [56]. Briefly, the cerebral cortices were dissected from the brains of mice, followed by the removal of the meninges and microvessels. The tissues were minced using sterile ophthalmic scissors and then digested with 0.05% trypsin at 37 °C for 10 min. The cell suspension was filtered through a 40 μm sieve, and the cells were subsequently seeded onto poly-D-lysine-coated 75 cm^2^ flasks with DMEM supplemented with 10% FBS, 100 U/mL penicillin, and 100 μg/mL streptomycin sulfate. The culture medium was refreshed on days 1 and 3. After 7 days, the microglia in the culture flasks were detached by shaking at 240 rpm for 3 h, while the remaining astrocytes were maintained in DMEM supplemented with 10% FBS. Experimental procedures were carried out following at least one passage of the cells.

Human glioma U251 cells were generously provided by Dr. Zejian Wang (Shanghai Jiao Tong University, Shanghai, China). The U251 cells were cultured in DMEM with 2 mM glutamine, 10% FBS, 100 U/mL penicillin, and 100 μg/mL streptomycin sulfate.

### 4.4. Immunofluorescence Staining and Image Analysis

Brain sections were rinsed with 0.05 M TBS, treated with 0.1% Triton X-100 in TBS for 10 min, and then incubated in 5% normal goat serum in TBS (0.1% Tween-20) for 30 min to block non-specific binding sites. For double immunofluorescence staining of CMKLR1 and GFAP or Aβ and GFAP, the sections were incubated for 1 h at room temperature or overnight at 4 °C with anti-CMKLR1/AF488 (1:200) or anti-Aβ (1:200) in TBS, respectively. After rinsing with TBS, the sections stained with anti-Aβ antibody were incubated with Alexa Fluor 647-conjugated anti-rabbit secondary antibody (1:500) at room temperature for 1 h. Then, the sections were incubated with anti-GFAP-Cy3™ antibody (1:200) in TBS for 1 h at room temperature. After washing in TBS, the sections were stained for nuclei with 100 ng/mL of DAPI for 10 min and mounted with 90% glycerol. The fluorescent confocal images were captured using a laser scanning confocal fluorescent microscope (TCS SP8, Leica Microsystems, Wetzlar, Germany). The expression of CMKLR1 and GFAP was quantified by analyzing immunofluorescence intensity using ImageJ/FIJI software 1.54f (NIH, Bethesda, MD, USA). The number of astrocytes (GFAP staining) colocalized with CMKLR1 or Aβ plaques (within 25 µm surrounding Aβ plaque [41]) was counted. Data are presented as the mean ± SEM based on 2 individual fields for each region, using three mice in each group.

To identify the expression of chemerin and its colocalization with microtubule-associated protein 2 (MAP2, neuron-specific molecule), brain sections were initially stained with anti-MAP2 (1:500) overnight. After three washes with TBS, the sections were incubated in Alexa Fluor 488-conjugated anti-rabbit secondary antibody (1:500) at room temperature for 1 h. The sections were rinsed in TBS and stained with anti-chemerin/AF647 (1:200) for 1 h at room temperature. Then, sections were counterstained with DAPI for 10 min and mounted with 90% glycerol. The fluorescent confocal images were taken on a laser scanning confocal microscope (A1 HD25, Nikon, Tokyo, Japan). Z-stack images were acquired using 20× water immersion objectives with a z-stack interval of 1.63 μm. The z-stack capture was performed by setting the bottom position at the appearance of the object of interest (first slice image) and the top position at the disappearance of the object of interest (last slice image). Merged maximum intensity projections (DAPI and chemerin) of the images were processed to quantify the expression of chemerin. The immunofluorescence intensity was analyzed using the ImageJ/FIJI software 1.54f. The colocalization analysis of chemerin in neurons stained with MAP2 was conducted using the Coloc2 plugin in ImageJ/FIJI software 1.54f. Pearson’s correlation coefficient was utilized to calculate the double fluorescence correlation coefficients. Scatter plots were employed to illustrate changes in the quantification of co-localized fluorescence [57]. Data are presented as the mean ± SEM based on 2 individual fields for each region, using three mice in each group.

### 4.5. Boyden Chamber Assay

Standard 48-well chemotaxis chambers (Neuro Probe, Gaithersburg, MD, USA) were used to detect the migration of astrocytes. The upper and lower wells of the chamber were separated by a polycarbonate membrane (10-μm pore size, Neuro Probe). The Boyden chamber assay was conducted as described previously [58]. Briefly, either DMEM or DMEM containing various concentrations of chemerin, C9, or 10% FBS were added to the lower wells of the chamber. The polycarbonate membrane was placed over the lower wells of the chamber. The upper chamber wells were filled with primary cultures of astrocytes or U251 cells with or without 15 min pretreatment of 1 μM C15. Following the addition of cells to the upper wells, the chamber was placed in a humidified incubator with 5% CO_2_ for 12 h. Subsequently, the filter was delicately extracted, and any non-migrating astrocytes on the upper surface were carefully removed using a cotton-tipped swab. The astrocytes that successfully migrated to the lower surface of the filter were fixed with methanol and stained with 0.1% crystal violet. Cell counting was performed using a phase-contrast inverted microscope (IX51, Olympus Optical Co., Ltd., Tokyo, Japan) equipped with a digital camera (EOS 1100D, Canon Inc., Tokyo, Japan). The results were quantified as the chemotaxis index, indicating the fold change in migrated cell numbers in response to the chemoattractant, compared to the control medium. Data are shown as the mean ± SEM from three independent experiments, each with three wells for each group.

### 4.6. Scratch-Wound Assay

The scratch-wound assay for evaluating cell mobility was performed [59]. Specifically, a scratch was created in confluent primary astrocytes or U251 cells cultured on coverslips precoated with poly-L-ornithine using a 10-μL pipette tip, resulting in the formation of a cell-free region measuring 500 μm in width. Subsequent movement of primary astrocytes and U251 cells was captured through light microscopy (Olympus Optical Co., Ltd.). Pictures were captured at 0 h as a control. The cells were incubated with chemerin (5 nM) with or without 15-min pretreatments with C15 for 8 h, 12 h, or 16 h. Then the pictures of the cells were taken at each time point, and the results were quantified by calculating the mean migrated distance of leading cells in the scratched area. Data are presented as the mean ± SEM from three independent experiments, each with three samples per group.

### 4.7. Assessment of Astrocyte Aggregation

Astrocyte aggregation was measured as described previously [60]. Briefly, the primary astrocyte cultures or U251 cells (1 × 10^4^) were seeded in a 24-well plate and incubated with 3 µM fibrillar Aβ_42_ with or without 5 nM chemerin or 100 nM C9 for 24 h. The cluster of astrocytes was defined as at least ten cells aggregated together around Aβ_42_. The distribution of aggregated astrocytes was captured with a phase contrast inverted microscope with a digital camera. The data are presented as the means ± SEM from three independent experiments, each with three wells for each group.

### 4.8. Western Blot

The tissues of the mouse brain were homogenized in lysis buffer containing 50 mM Tris-HCL (pH 7.4), 2 mM EDTA, 100 mM NaF, 2 mM sodium vanadate, 10 mM β-mercaptoethanol, 8.5% sucrose, 100 µg/mL leupeptin, 5 µg/mL aprotinin, and 5 µg/mL pepstatin. Protein concentrations were validated using BCA kits. The samples were mixed with 5× sodium dodecyl sulfate (SDS)-PAGE loading buffer, heated at 99 °C for 10 min, and separated by 10% or 12% SDS-PAGE. Subsequently, the proteins were transferred to nitrocellulose membranes (GE Healthcare, Wauwatosa, WI, USA). The membranes were blocked for 1 h at room temperature with 5% non-fat milk and then incubated at 4 °C overnight with anti-STING (1:1000), anti-phospho-STING (1:1000), or anti-β-tubulin (1:1000) antibodies, followed with corresponding HRP-labeled IgG secondary antibodies. The membranes were visualized using a Super ECL Plus (US Everbright, Suzhou, China) under the ChemiDoc MP Imaging System (Bio-Rad, Richmond, CA, USA). Densitometric quantification of protein bands was determined using the ImageJ software 1.54f (NIH, Bethesda, MD, USA).

### 4.9. Statistical Analyses

The data are shown as means ± SEM. Two-group comparisons were assessed using the two-tailed *t*-test. For multiple comparisons, one-way ANOVA with Tukey’s post hoc test was employed. Statistical analyses were conducted using GraphPad Prism 8 (San Diego, CA, USA). A *p*-value smaller than 0.05 was considered statistically significant. 

## Data Availability

All datasets generated or analyzed during this study are available from the corresponding author on reasonable request.

## Supplementary Materials

The following supporting information can be downloaded at: https://www.mdpi.com/article/10.3390/ijms25084324/s1.
