# Supplementary material for: Effect of Regulation of Chemerin/Chemokine-like Receptor 1/Stimulator of Interferon Genes Pathway on Astrocyte Recruitment to Aβ Plaques
Source: Int J Mol Sci. 2024 Apr 13;25(8):4324. doi: 10.3390/ijms25084324 (PMC11049903; doi:10.3390/ijms25084324)
Supplement: Supplementary file 1 [file ijms-25-04324-s001.zip › ijms-2924100-supplementary.pdf]

SUPPLEMENTARY MATERIALS

Supplementary methods:

Cell viability measurement

Cell viability was assessed by a methyl thiazolyl tetrazolium (MTT) colorimetric assay. Primary cultures of astrocytes or U251 cells in 96-well plates were exposed to chemerin (20 nM) or C9 (100 nM) for 16 h. Following treatment, the MTT reagent (1 mg/ml) was added and the cells were incubated at 37 °C for 4 h. Subsequently, the formazan crystals were solubilized in dimethyl sulfoxide after three washes with phosphate-buffered saline (PBS, pH 7.4). The absorbance at 570 nm was measured using a FlexStation III plate reader (Molecular Devices, Mountain View, CA). Cell viability was calculated as a percentage relative to the control.

Supplementary figures

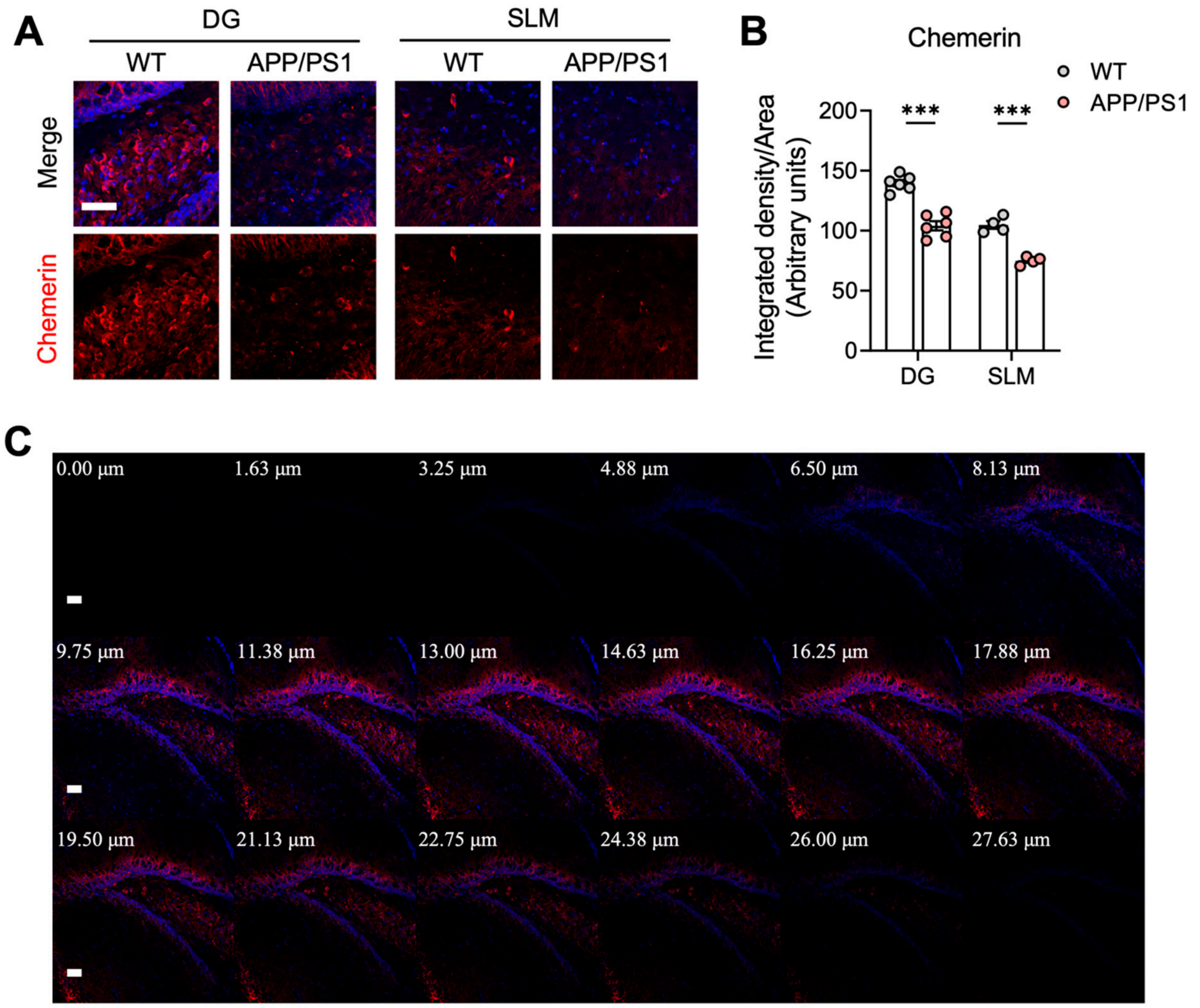

Supplementary Figure S1. Reduced chemerin expression in the hippocampus of APP/PS1 transgenic mice. (**A**) Immunofluorescence staining was conducted to detect the expression of chemerin. Serial sections in the DG and SLM regions of the hippocampus from 9-month-old WT and APP/PS1 mice were stained for chemerin using a rabbit anti-chemerin/AF647 antibody (red fluorescence). Cell nuclei were stained with DAPI (blue fluorescence). Z-stack confocal images were acquired and merged into maximum intensity projections (chemerin and DAPI). Scale bar, 50 μm. (**B**) Quantification of the fluorescence intensity of chemerin is shown. Data are shown as the mean ± SEM based on 2 individual fields for each region, using three mice in each group. *** $p < 0.001$. (**C**) Representative Z-stack series with 1.63-μm intervals in the z-direction were captured from the brain of a 9-month-old WT mouse. Scale bar, 50 μm. DG: Dentate Gyrus, SLM: Stratum Lacunosum-Moleculare.

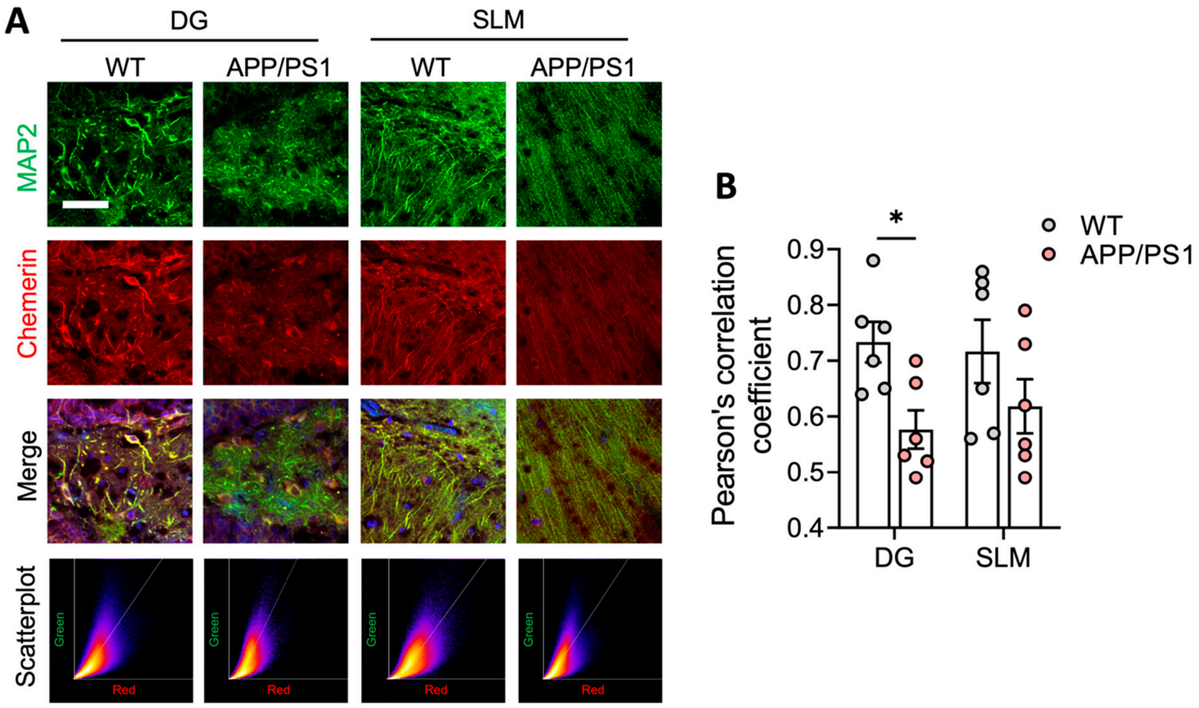

Supplementary Figure S2. Downregulation of chemerin and neuronal colocalization in the hippocampus of APP/PS1 transgenic mice. (**A**) Immunofluorescence staining was conducted to detect the expression of chemerin in neurons. Serial sections in the DG and SLM regions of the hippocampus from 9-month-old WT and APP/PS1 mice were stained for chemerin using a rabbit anti-chemerin/AF647 antibody (red fluorescence) and neuronal marker MAP2 using a rabbit anti-MAP2 polyclonal antibody and Alexa fluor 488-conjugated anti-rabbit IgG (green fluorescence). Cell nuclei were stained with DAPI (blue fluorescence). Representative scatterplots showing pixel intensities in the red channel versus the green channel are displayed below each merged image, with Pearson's correlation coefficients of r = 0.77, 0.66 in the DG region of WT and APP/PS1 mice, respectively, and 0.82, 0.73 in the SLM region. Scale bar, 50 μm. (**B**) Pearson's correlation coefficient was utilized to quantify the colocalization of chemerin and MAP2. ImageJ/Fiji software 1.54f was used to calculate the coefficient. Data are shown as the mean ± SEM based on 2 individual fields for each region, using three mice in each group. * $p < 0.05$. DG: Dentate Gyrus, SLM: Stratum Lacunosum-Moleculare.

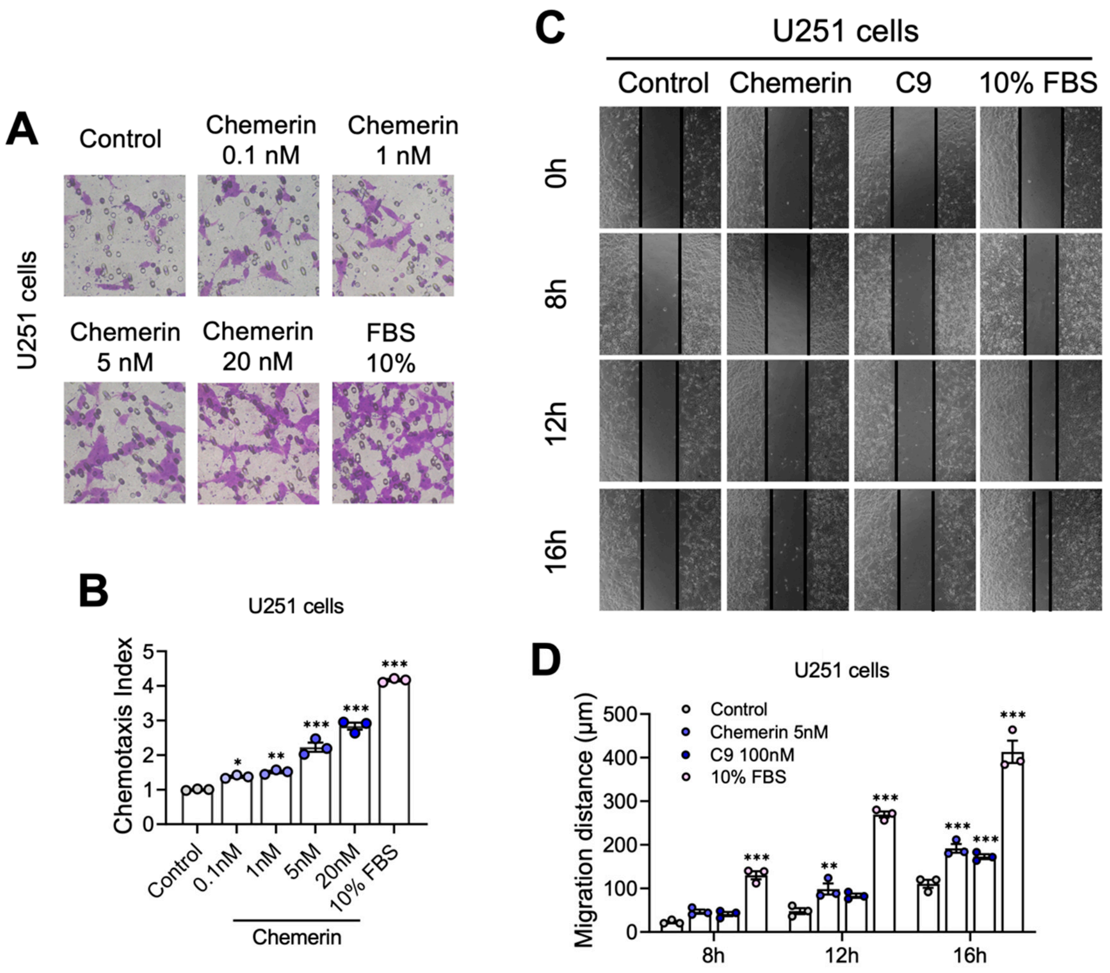

Supplementary Figure S3. Chemerin induces the migration of U251 cells. (**A**) The Boyden chamber migration assay was conducted to determine cell migration. U251 cells were treated with chemerin (0.1-20 nM) or 10% FBS for 12 h. Representative images of migrated cells on membrane filters are shown. Magnification, 400×. (**B**) Quantification of the fold increase in the number of migrated cells in response to chemerin or 10% FBS over the control medium was shown. (**C**) U251 cells were treated with 5 nM chemerin, 100 nM C9, or 10% FBS, and cell migration was detected by the scratch-wound assay. The cells were photographed at 0 h, 8 h, 12 h, and 16 h. Representative images of migrated cells are shown. Magnification, 100×. (**D**) Quantification of the migration distance is shown. Results are expressed as the mean ± SEM based on three independent experiments, each in triplicate. * $p < 0.05$, ** $p < 0.01$, *** $p < 0.001$.

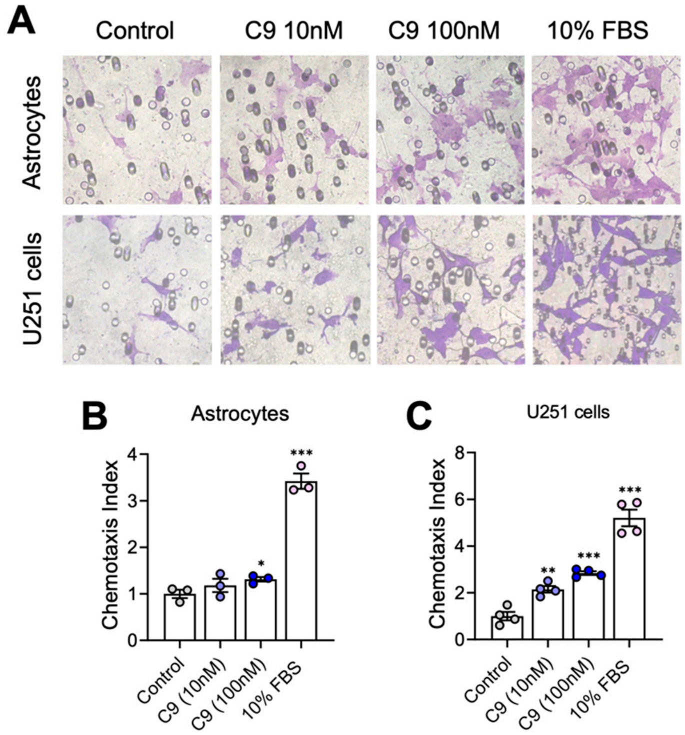

Supplementary Figure S4. C9 induces the migration of primary cultures of astrocytes and U251 cells. (**A**) The Boyden chamber migration assay was conducted to determine cell migration. Primary astrocytes and U251 cells were treated with C9 (10 nM and 100 nM) or 10% FBS for 12 h. Representative images of migrated cells on membrane filters are shown. Magnification, 400×. (**B, C**) Quantification of the fold increase in the number of migrated cells in response to C9 or 10% FBS over the control medium is shown. The results are expressed as the mean ± SEM from three separate experiments, each in triplicate. *$p < 0.05$, **$p < 0.01$, ***$p < 0.001$.

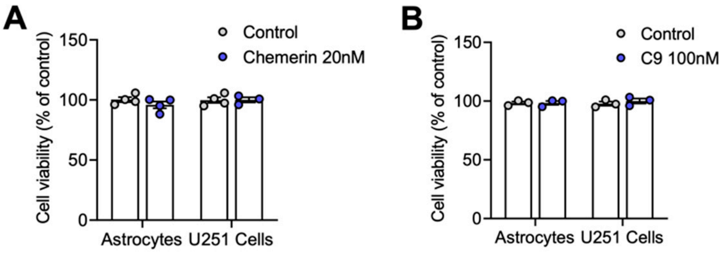

Supplementary Figure S5. Chemerin and C9 do not affect the cell viability of primary astrocytes and U251 cells. (**A, B**) Primary cultures of astrocytes and U251 cells were treated with chemerin (20 nM) or C9 (100 nM) for 16 h. MTT assay was conducted to test the viability of the cells. Quantification of the percentage of surviving cells over control cells is shown. Data are presented as the mean ± SEM based on three independent experiments, each in triplicate.
